# Association of Psychological Resilience with All-Cause and Cardiovascular Mortality in a General Population in Italy: Prospective Findings from the Moli-Sani Study

**DOI:** 10.3390/ijerph19010222

**Published:** 2021-12-25

**Authors:** Anwal Ghulam, Marialaura Bonaccio, Simona Costanzo, Alessandro Gialluisi, Federica Santonastaso, Augusto Di Castelnuovo, Chiara Cerletti, Maria Benedetta Donati, Giovanni de Gaetano, Francesco Gianfagna, Licia Iacoviello, on behalf of the Moli-Sani Study Investigators

**Affiliations:** 1 Department of Medicine and Surgery, Research Center in Epidemiology and Preventive Medicine (EPIMED), University of Insubria, 21100 Varese, Italy; ghulam.anwal@uninsubria.it (A.G.); federica.santonastaso95@gmail.com (F.S.); francesco.gianfagna@uninsubria.it (F.G.); licia.iacoviello@moli-sani.org (L.I.); 2 Department of Epidemiology and Prevention, IRCCS NEUROMED, 86077 Pozzilli, Italy; simona.costanzo@moli-sani.org (S.C.); alessandro.gialluisi@moli-sani.org (A.G.); chiara.cerletti@moli-sani.org (C.C.); mbdonati@moli-sani.org (M.B.D.); giovanni.degaetano@moli-sani.org (G.d.G.); 3 Mediterranea Cardiocentro, 80121 Napoli, Italy; dicastel@ngi.it

**Keywords:** psychological resilience, mortality, cardiovascular mortality, population study

## Abstract

Psychological resilience (PR) is the capacity to adapt positively in face of adversity. Its role as an independent protective factor has been acknowledged in recent years. We aimed to test the association of PR with all-cause and cardiovascular disease (CVD) mortality in a general adult population. We performed longitudinal analyses on 10,406 CVD-free individuals from the Moli-Sani cohort (follow up = 11.2 year). PR was assessed by the 25-item Connor and Davidson resilience scale. PR factors were identified through polychoric factor analysis. Associations with mortality were tested using multivariable Cox regressions. Higher levels of PR were associated with reduced all-cause mortality in a model including sex and age (HR = 0.78; 95%CI 0.62–1.00). The association decreased after inclusion of socioeconomic, clinical, and behavioral factors into the model (HR = 0.80; 95%CI 0.62–1.03). No relation was observed with cardiovascular mortality in the fully adjusted model (HR = 0.89; 95%CI 0.56–1.39). An inverse association of Factor 1 (reflecting positive acceptance of change) with all-cause mortality (HR = 0.89; 95%CI 0.82–0.98; *p* value = 0.01) was found. However, at a borderline non-significant way, PR predicts all-cause mortality in a general population of Italian adults. This is supported by the findings demonstrating a significant association between the PR’s domain reflecting a positive acceptance of change and all-cause mortality.

## 1. Introduction

Cardiovascular disease (CVD) remains the leading cause of death and disease burden globally [1]; in fact, in Europe alone 45% of deaths are attributable to CVD [2]. Despite the remarkable advancements made in the last century, classical risk factors explain only part of CVD incidence. Moreover, much of this research has been conducted from a *pathogenesis* perspective, which has been the centre of medical research for centuries. In the epidemiological field this research has been translated in the enquiry of risk factors that promote illnesses and mortality. However, there have been important ramifications in the field, including health promotion [3].

A compelling theory of health promotion is the *Salutogenic* orientation, which focuses its attention on *salutary factors*, i.e., potential factors that can promote health instead of disease [4]. In this framework, many have investigated psychosocial exposures as potential protective factors from illnesses, for instance concepts like optimism [5,6], sense of coherence [7,8], and psychological resilience (PR) [9] have shown encouraging evidence of their independent protective effect on health, yet, not all research has found such psychological exposures to be protective [10]. 

In particular, PR can be defined as the capacity of individuals to cope successfully with significant change, adversity, or risk [11]. Initially PR was conceptualised as a trait [12], but with time the understanding of PR as dynamic process became more predominant. When considered as a process, PR can change with time, context, age, gender, and cultural origin as well as within an individual subject to different life circumstances [13]. Measurement of PR is a challenging task and researchers have devised many tools, including self-administered questionnaires. In two different systematic reviews, the Connor and Davidson resilience scale was classified as having good-enough psychometric qualities, and thus scored among the most appropriate options [14,15]. 

PR research flourished in psychiatric settings, and questionnaires were initially designed as outcome measurements for psychiatric patients, in particular patients that had suffered trauma. PR research in non-psychiatric/psychologic setting is in its infancy and analyses are mostly explorative. There are only three prospective cohort studies in our knowledge that have investigated PR’s association with cardiovascular health and survival with a longitudinal design. The Swedish military conscription cohort found PR to be associated with lower incidence of stroke, coronary heart diseases, hypertension, diabetes, and heart failure [9,16,17,18,19]. A sub-cohort of African-American women from the Women’s Health Initiative study found no association between PR and CVD risk [10]. Lastly, the Chinese Longitudinal Healthy Longevity Survey reported evidence of an association of PR with decreased mortality in elderly subjects (>65 years) [20]. However, none of these studies evaluated hard endpoints such as total CVD mortality in a general population of adults.

To fill this knowledge gap, we used data from the general population of the Moli-Sani Study cohort (Italy) and tested the hypothesis that higher PR levels in the population are associated with a lower risk of all-cause and CVD mortality. However, we also acknowledge an important limitation from the very beginning: PR nature in general populations may have a different impact on overall health compared to psychiatric populations. General populations most probably have experienced a lesser amount of stress and adversity than their psychiatric counterparts. 

## 2. Materials and Methods

### 2.1. Study Population 

The Moli-Sani Study is a prospective cohort study of 24,325 men and women randomly recruited from the general population of Molise, a Southern region of Italy. The cohort was established between March 2005 and April 2010 with the main aim of investigating genetic and environmental determinants of non-communicable chronic diseases. Exclusion criteria were pregnancy at the time of recruitment, mental disturbances or decision-making impairments, current poly-traumas or coma, or refusal to sign the informed consent. The Moli-Sani Study was approved by the Ethics Committee of the Università Cattolica del Sacro Cuore (Rome, Italy) and all participants provided written informed consent. Details of the study have been provided in full elsewhere [21]. Assessments of exposure and covariates were made at the baseline only.

Of the original cohort of 24,325, a random sample of 18,680 participants underwent a psychological examination, of which 11,702 (63%) provided complete information on PR, and thus were included in the present analysis. After further exclusion of subjects with any history of cardiovascular disease (*n* = 508), missing data on CVD (*n* = 97), unreliable medical or dietary questionnaires as judged by interviewers (*n* = 341), and missing data on CVD mortality (*n* = 9), 10,406 subjects were included in the final analysis.

The analysed individuals (*n* = 10,406), compared to those that underwent psychological examination but were excluded from the present study (*n* = 6251), were prevalently men, younger, and had lower cumulative socioeconomic disadvantage (CSD). No significant differences were observed for the prevalence of major health conditions nor health behaviours between the two groups (Supplementary Table S1). 

### 2.2. Psychological Resilience Assessment

PR was assessed at baseline through the Connor–Davidson Resilience Scale (CD-RISC) [13] which was developed as a self-rate assessment to measure PR; the content of the scale is drawn from various sources of resilience literature, but the items mainly pertain to domains of personal competence, trust/tolerance/strengthening effects of stress, acceptance of change, secure relationships, control, humour, patience, and spiritual influences. The CD-RISC consists of 25 items, which are rated on a five-point Likert scale and range from 0 (“Not true at all”) to 4 (“True nearly all the time”), and the total PR score ranges from 0 to 100, with higher values reflecting higher levels of PR. For analyses purposes, we ranked PR score into quartiles, or used the score as a continuous variable for 1 standard deviation (SD) increase. 

### 2.3. Vital Status Ascertainment 

The Moli-Sani Study cohort was followed up for mortality until 31 December 2018. Cause-specific mortality was assessed by the Italian mortality registry, validated by Italian death certificates (ISTAT form) and coded according to the International Classification of Diseases (ICD-9). 

CVD mortality included deaths from diseases of the circulatory system when the underlying cause of death included ICD9 codes 390–459. 

### 2.4. Assessment of Covariates

#### 2.4.1. History of Diseases and Behavioural Factors

History of cardiovascular disease (angina, myocardial infarction, peripheral artery disease, revascularisation procedures, and cerebrovascular events) was self-reported and confirmed by medical records and therapy. History of cancer was self-reported and confirmed by medical records. Participants were expected to have diabetes, hypertension, or hyperlipidaemia if they were receiving disease-specific drugs. Leisure-time physical activity (PA) was expressed as daily energy expenditure in metabolic equivalent task-hours (MET-h/d) for sport, walking, and gardening. Height and weight were measured, and body mass index (BMI) was calculated as kg/m^2^. Subjects were classified as never-smokers, current, or former smokers (quit at least 1 year ago). 

#### 2.4.2. Socioeconomic Status

Cumulative socioeconomic disadvantage (CSD) over life-course was assessed by a score computed using three childhood socioeconomic factors [22]: (a) availability of hot water (coded 0-1); (b) overcrowding and housing tenure (both coded 0-1); (c) 3-level educational attainment (coded 0-1-2 with none/primary and lower secondary collapsed in the same category); three adulthood socioeconomic status (SES) indicators (housing and occupational class were coded 0-1-2, and overcrowding was coded 0-1). The final score ranged from 0 to 10 with the highest values indicating the greatest CSD. Marital status was considered as married/cohabiting, divorced/separated, and single or widowed. 

#### 2.4.3. Dietary and Psychological Assessment

The Mediterranean Diet Score, developed by Trichopoulou et al., was used to estimate diet quality as reflected by adherence to the Mediterranean diet [23]. Psychological assessment at baseline was categorised into the following five mutually exclusive groups: (a) use of anti-depressive drugs at least once in their life; (b) use of psychoactive drugs other than antidepressants at least once in their life; (c) self-reported previous diagnosis of depression or anxiety or insomnia with no use of psychoactive drugs; (d) psychologically healthy subjects (individuals who failed to be in categories a, b or c); and (e) missing information (individuals with missing data on previous diagnosis of psychological disease or use of psychoactive drugs).

Quality of life was assessed by using the validated Italian version of the self-administered SF-36 [24]. 

### 2.5. Statistical Analysis 

Analyses were performed on 10,406 individuals that were grouped in quartiles of PR with 2600 subjects in each quartile. Prior data showed that the rate of events (total mortality) was 0.05. 

Under the assumption of a 0.05 rate of all-cause mortality events—suggested by previous studies in the Moli-Sani cohort—we were able to detect true hazard ratios (≤0.70 or ≥1.37) in one group in comparison with another, with probability (power) 0.8, at a Type I error probability α = 0.05.

For CVD mortality (expected rate 0.015), we were able to detect significant hazard ratios (α = 0.05) ≤0.46 or ≥1.73.

Baseline characteristics of the participants according to quartiles of PR are represented as means with standard deviation (±SD) or number and percentages for continuous and categorical values, respectively. Differences in the distribution of baseline covariates according to PR quartiles were calculated using generalised linear models adjusted for age, sex, and energy intake (GENMOD procedure for categorical variables and GLM procedure for continuous variables in SAS software).

The proportional hazards assumption was assessed using weighed Schoenfeld residuals, and no violation was identified (*p* value for global test = 0.12).

Rate estimates for all-cause and CVD mortality were expressed as hazard ratios (HRs) with 95% confidence intervals (95%CI), and calculated by using Cox proportional hazards models with time-on-study on the time scale and adjusting for baseline age as covariate in the model. Multivariable-adjusted HRs were calculated across quartiles of PR, as well as considering PR as a continuous variable (1 SD increase).

Participants contributed person-time until date of death, date of emigration or loss-to-follow-up, or end of follow-up, whichever occurred first. Participants who died from another cause than the one under study were included and censored at the date of the competing death event. 

In addition to a crude model, we used a Model 1 adjusted for age (continuous) and sex, which was alternately controlled for the following set of factors: (a) Model 1 + clinical factors at baseline (history of cancer, diabetes, hypertension, hypercholesterolemia, and psychological status); (b) Model 1 + behavioural factors (smoking, physical exercise, adherence to Mediterranean diet, and body mass index); (c) Model 1 + socioeconomic/social factors (CSD, place of residence, and marital status); and (d) all factors included simultaneously into the model. 

Distribution of missing values was as follows: CSD (*n* = 402), leisure-time physical activity (*n* = 78), BMI (*n* = 2), smoking habit (*n* = 6), history of cancer (*n* = 27), diabetes (*n* = 106), hyperlipidaemia (*n* = 58), hypertension (*n* = 52), and psychological assessment (*n* = 47). We used a multiple imputation technique (SAS PROC MI, followed by PROC MIANALYZE) to maximise data availability for all variables, avoid bias introduced by not-at-random missing (MNAR) data patterns, and achieve robust results over different simulations (*n* = 10 imputed datasets). Statistical tests were two-sided, and Bonferroni correction for multiple comparisons was applied to each analysis according to the number of independent tests carried out (see Tables). Data analyses were generated using SAS/STAT software, version 9.4 (SAS Institute Inc., Cary, NC, USA).

A polychoric factor analysis (PFA) was performed through the *psych* package in R (https://www.r-project.org/; accessed 1 October 2021) to infer a reduced number of latent variables from the 25 psychological resilience items assessed through the CD-RISC [25]. This was aimed at identifying different domains of psychological resilience—each tagged by a polychoric factor—to better disentangle the association between PR and mortality. After computing a polychoric correlation matrix, five main factors were inferred according to their eigenvalues being greater than 1 (8.30, 1.73, 1.31, 1.21, and 1.05 respectively). Then, a final PFA was computed by applying a *varimax* (orthogonal) rotation to our population sample (N = 10,406), to compute the final factor scores and assess the relevant loadings of each PR item.

## 3. Results

The analysed sample consisted of 5236 women (50.3%) and 5170 men (49.7%), with a mean age of 52.1 year (SD = 10.5; min–max 34.6–91.1 year) and an average PR score of 66.8 (SD = 12.4; interquartile range 59–75).

People with higher PR were younger, had lower CSD, prevalently lived in urban areas, and were married. Resilient people were more likely to exhibit healthier behaviours, as reflected by higher levels of physical activity and an increased adherence to a Mediterranean diet. Resilient people had also less prevalence of hypertension, lower BMI, and psychological disease (Table 1).

Over a median follow-up of 11.2 years (interquartile ranges 10.2–12.1 year), 478 all-cause and 140 CVD deaths were recorded. Cox regression analysis showed that in the model controlled for age and sex (model 1), subjects in the highest quartile of PR were at 22% lower risk of all-cause death (HR = 0.78; 95%CI 0.62–1.00; *p* for trend = 0.029) compared to the first quartile. This association was still significant after adjustment for baseline health conditions, including history of cancer, diabetes, hypertension, hyperlipidaemia, and psychological status (HR = 0.76; 95%CI 0.59–0.97; *p* for trend = 0.017). Inclusion of baseline health behaviours into Model 1 slightly attenuated the association of PR (Q4) with all-cause death risk (HR = 0.79; 95%CI 0.62–1.01; *p* for trend = 0.040), and was further reduced when CSD, social factors, and residence were entered into model 1 (HR = 0.83; 95%CI 0.64–1.06; *p* for trend = 0.087). Inclusion of all factors into the model yielded an HR of 0.80 (95%CI 0.62–1.03; *p* for trend = 0.059). There was no significant association between PR and CVD mortality except in the crude model (HR = 0.62; 95%CI 0.40–0.96 for Q4 vs. Q1; *p* for trend = 0.031) (Table 2).

We performed an additional Cox regression to see whether factors identified by polychoric factor analysis and tagging different domains of psychological resilience were associated with CVD and all-cause mortality in our study population. We identified five factors that we defined in the following way: Factor 1 was related to positive acceptance of change (items 1, 4, 5, 6, 7, and 8); Factor 2 was related to faith and hope (items 3, 9, 20, and 21); Factor 3 was related to secure attachment and secure relationships (items 2 and 13); Factor 4 was related to external locus of control and confidence in one’s abilities (items 11, 22, 24, and 25); and Factor 5 was related to strengthening effects of stress and self-efficacy (items 12, 15, 16, 17, and 18) (Supplementary Table S2). In a multivariable-adjusted Cox regression, Factor 1, which reflected positive acceptance of change, was associated with an 11% lowering of all-cause mortality risk (HR = 0.89 95%CI 0.82–0.98: *p* value = 0.01) for each SD increment in Factor 1 (Table 3).

The same inverse trend of Factor 1 was observed in association with CVD mortality, although statistical significance was not retained (HR = 0.88; 95%CI 0.74–1.03; *p* value = 0.11).

In sub-group analyses (Table 4), we observed an effect modification of age, since a 1-SD increase in PR was associated with an increased risk of CVD mortality among participants aged ≤ 65 years (HR = 1.40; 95%CI 1.02–1.93), while a decreased risk in the group aged > 65 years was found (HR = 0.88; 95%CI 0.74–1.04; *p* for interaction = 0.036). No other substantial sub-group differences were documented, neither in the analysis of PR nor in the stratified analysis of polychoric factors (Supplementary Table S3).

## 4. Discussion

In this cohort of 10,406 CVD-free individuals from the general population of the Moli-Sani Study, an increase in the capacity of adapting to change (Factor 1) was associated with a lower risk of all-cause mortality. Overall, results point to a 20% decreased risk of all-cause mortality associated with higher PR, although multivariable adjustment did not retain formal statistical significance. For CVD death risk, the association with PR followed a similar downward trend, without reaching statistical significance. In line with our research hypothesis, baseline PR levels were slightly able to predict mortality in this cohort of 10,406 adults from a general Italian population of adults.

The role of PR as an independent predictor of mortality and diseases has not been extensively investigated so far, and the available prospective studies provided mixed results. In particular, the Swedish military conscription cohort, with numerosity ranging from 237,980 [9,16] to 1,784,450 [19], found lower PR to be consistently associated with increased disease incidence, namely stroke [9], coronary heart diseases [16], hypertension [17], type 2 diabetes [18], heart failure [19], inflammatory bowel disease [26], cancer [27], and peptic ulcer disease [28]. The cohort enrolled all Swedish young men (18–20 years) in the years that military conscription was obligatory. PR in this cohort was assessed through a semi-structured interview with a psychologist, that had the aim to assess the capabilities of the recruited subject to withstand military stresses. Because of military secrecy, questions are not available to the public, however, it is reported that the assessed domains concerned adjustment problems and conflicts, successes, responsibilities taken on, and initiatives shown or experienced in school, work, home, or in leisure activities. Furthermore, emotional stability, social maturity, and active/passive interests were also rated by the psychologist, who then assigned a summary score on a standardised nine scale (one to nine) [17]. The main limitation of this study is provided by the unique military young male cohort of Europeans. Additionally, PR assessment in this cohort may not reflect the current meaning of PR because of chronological reasons. The semi-structured interview used in the military conscription has been the same, to our knowledge, from the 1960s, and even though PR literature dates back to the 1960s–1970s, it did not have a complete conceptual analysis nor further operationalisation, (i.e., transformation of the construct in measurable form, in this case questionnaire), until the end of the 1980s [12].

The second prospective study comes from a cohort of 2765 black American women of post-menopausal age. Results showed no association between CVD events and PR. PR was measured through a modified version of the Brief Resilience Scale (three items) [29]. The biggest limitation of this study is that results are not generalisable because of the gender and the ethnicity of the subjects. The third prospective study derives its data from the Chinese Longitudinal Health Longevity survey that included 13,800 elderly subjects (>65 years) and found lower PR to be associated with mortality [20]. PR in this study was assessed through a modified version of the Connor and Davidson Resilience Scale, that has been shown to have good psychometric properties in a sample of Chinese subjects [30], and subjects were followed up for 3 years. 

Among these three cohorts, the Chinese one resembles our cohort better because it presents both genders from the general population, although PR was assessed with a modified version of the CD-RISC in an ethnically different group compared to the Moli-Sani cohort. 

Moreover, in cross-sectional studies, resilient populations were found to have an association with better lipid profiles [31,32], better HbA1c levels [33], and better metabolic syndrome status [34]; on the other hand, results on the association of resilience with BMI were not consistent, with some studies showing an inverse relationship [32] and others showing none [35,36,37,38]. These cross-sectional studies also confirmed the results from prospective studies and showed that resilient populations had better diets, healthier lifestyle habits, were more physically active, and had higher SES [9,10,16,36]. The fact that PR is associated with health factors that are on their own associated with better health explains why, after adjustment for these factors, the association of PR with mortality slightly lost power, indicating that these factors possibly explain part of the observed relationship.

A peculiar characteristic of our study is that we identified individual domains of PR that were then related to the outcomes. This analysis allowed us to see the patterns of item correlation in our cohort, which is very similar to the one found in the original studied population by Connor and Davidson [13]. Factor 1, which is related to a positive acceptance of change, was associated with lower all-cause mortality overall and led to lower all-cause and CVD death estimates among elderly participants. Positive acceptance of change is at the core of PR, i.e., it is only when to adversity follows a positive adaptation that PR has a motive to manifest, and accepting the change as positive allows the possibility to react positively [39]. As stated above, PR differs in different cultures [40], thus, the understanding of PR in south-Italian subjects could be different from the resilient characteristics identified by Connor and Davidson that subsequently were made part of the questionnaire. Perhaps a positive acceptance of change, and the capability to face the future and unexpected events with a positive attitude, is the strongest resilience trait in Italian subjects, and thus, the only one able to predict mortality. 

### Strengths and Limitations 

To the best of our knowledge, this is the first study that investigated the association between PR and mortality for all-cause and CVD mortality in a general population, and used a broad range of variables as adjustment factors; furthermore, we performed a factor analysis of the different PR items and a subsequent Cox regression with the identified factors. This study has significant strengths, including its prospective design, the relatively long follow-up of over 10 years, and the detailed information on several lifestyle and clinical factors to minimise confounding. PR was assessed through one of the better psychometrically and conceptually performing questionnaires in two systematic reviews [14,15].

Despite the abovementioned strengths, this study has several limitations. A drawback of observational studies is that causality cannot be inferred and even though analyses in this study were controlled for several factors, we cannot fully rule out the potential of residual confounding by unmeasured factors. All data were collected at baseline only, thus changes which possibly occurred over the course of life might have modified the strength of the findings. The CD-RISC, by account of being a questionnaire, is unable to capture the dynamic nature of PR (like most other assessment tools), and due to the loss of the dynamic aspect, our results may be showing just part of the effect that PR may have on health and illness. Additionally, our study design did not include an assessment of traumatic life events that could have better informed the results of the PR score in our population. It is important to mention that PR was not originally designed to be used in a non-psychiatric setting, although several studies have used it outside of psychiatric populations (e.g., [33,41,42]). Thus, the nature of PR and its impact on health in general populations may differ significantly from psychiatric populations because psychiatric patients most probably have suffered more adverse events and trauma. Finally, data were gathered within an Italian sample, thus generalisability to other contexts should be made with caution.

## 5. Conclusions

Our study in a large sample of Italian adults shows that, even though in a marginally non-significant way, PR score is associated with all-cause mortality. This conclusion was supported by the finding that Factor 1 (reflecting positive acceptance of change) was associated with a significant decreased risk of all-cause mortality. 

In a health-promotion framework, finding an association between PR and hard outcomes with mortality is a compelling task. Our findings would give a reason to dig deeper into what makes people resilient, and to find ways of promoting it. In our cohort, the factor associated with positive acceptance of change was associated with lower all-cause mortality; thus, educating subjects to accept change positively would have been an effective health-promotion intervention in this cohort. However, PR differs across cultures and studying PR without knowledge of the adversities the subjects may have faced can only give a snapshot of the effect of PR on health. Future research should consider the different understandings of PR and the different burdens of adversity that their specific populations may have. Thus, to overcome at least some of the limitations of our study, it would be advisable to assess traumatic life events and repeat a PR measurement many times throughout the follow up.

## Figures and Tables

**Table 1:** Baseline characteristics of the study population (*n* = 10,406) according to psychological resilience at baseline (2005–2010), as measured by Connor–Davidson Resilience Scale (CD-RISC).

	Psychological Resilience				
	Q1	Q2	Q3	Q4	
CD-RISC (median, IQR)	53 (48–56)	63 (61–65)	70 (68–72)	81 (77–86)	*p*-value
Number of subjects (%)	2473 (23.8)	2647 (25.4)	2583 (24.8)	2703 (25.9)	-
Age (y; means ± SD)	53 (11)	52 (10)	51 (10)	52 (10)	<0.0001
Men	46.1	50.9	51.1	50.4	0.0006
Urban residence	63.1	66.5	67.7	70.5	<0.0001
Marital status					<0.0023
Married/cohabiting	84.4	87.3	88.4	87.6	
Separated/divorced	2.6	2.9	3.1	3.5	
Single	6.1	5.9	5.0	5.2	
Widower	6.9	3.9	3.5	3.6	
Cumulative disadvantage score (means ± SD)	5.6 (1.8)	5.2 (1.8)	5.0 (1.7)	4.8 (1.8)	<0.0001
Physical exercise (MET-h/d; means ± SD)	3.1 (3.6)	3.5 (3.8)	3.5 (3.8)	3.6 (3.8)	<0.0001
Mediterranean diet score (means ± SD)	4.2 (1.7)	4.3 (1.7)	4.4 (1.6)	4.4 (1.7)	<0.0001
Body mass index (means ± SD)	27.8 (4.6)	27.4 (4.5)	27.4 (4.5)	27.8 (4.6)	0.010
Smoking status					0.33
Non- smokers	49.8	47.6	47.9	47.8	
Current	24.4	26.0	24.3	23.2	
Former	25.7	26.4	27.7	28.9	
Missing data	0.08	0.04	0.04	0.07	
Cancer					<0.0001
No	97.1	97.8	97.4	95.6	
Yes	2.6	2.2	2.1	4.1	
Missing data	0.3	0.04	0.5	0.2	
Diabetes					0.089
No	94.6	95.6	96.5	96.4	
Yes	4.4	3.3	2.4	2.8	
Missing data	1.1	1.1	1.1	0.8	
Hyperlipidaemia					0.58
No	93.6	95.3	95.7	94.9	
Yes	5.5	4.2	3.7	4.6	
Missing data	0.7	0.5	0.6	0.5	
Hypertension					<0.0001
No	75.2	79.0	79.56	78.7	
Yes	23.3	20.3	19.3	20.5	
Missing data	0.7	0.5	0.5	0.3	
Psychological assessment					<0.0001
Psychologically healthy	82.7	89.0	89.9	91.0	
Antidepressant use	4.5	3.0	2.4	2.1	
Use of psychoactive drugs	6.5	4.0	3.4	3.2	
Self-reported diagnosis of psychological disease	5.7	3.6	3.8	3.3	
Missing data	0.6	0.3	0.5	0.4	

Values are expressed as percentages unless otherwise stated. *p* values are adjusted for age and sex.

**Table 2:** Hazard ratios (with 95% confidence interval) for all-cause and cardiovascular mortality associated with psychological resilience (quartiles) in the Moli-Sani Study cohort (*n* = 10,406) using data obtained from multiple imputation.

	Psychological Resilience (PR) Quartiles					
	Q1	Q2	Q3	Q4	*p*-Value for Trend	1-SD Increment in PR
**All-cause mortality (*n* = 478)**						
N of events/*n* of subjects	159/2473	115/2647	93/2583	111/2703	-	-
Person-years	27,204	29,341	28,721	29,851	-	-
Crude model	-1-	0.67 (0.53–0.85)	0.55 (0.43–0.71)	0.64 (0.50–0.81)	<0.0001	0.83 (0.76–0.91)
Model 1 (age, sex)	-1-	0.85 (0.67–1.08)	0.75 (0.58–0.97)	0.78 (0.62–1.00)	0.029	0.93 (0.86–1.01)
Model 1 + baseline disease/conditions	-1-	0.85 (0.67–1.08)	0.76 (0.59–0.99)	0.76 (0.59–0.97)	0.017	0.92 (0.85–1.00)
Model 1 + health behaviors	-1-	0.85 (0.67–1.08)	0.76 (0.59–0.99)	0.79 (0.62–1.01)	0.040	0.94 (0.86–1.02)
Model 1 + CSD, social factors and residence	-1-	0.88 (0.69–1.12)	0.79 (0.61–1.03)	0.83 (0.64–1.06)	0.087	0.95 (0.87–1.03)
Model 1 + all factors	-1-	0.88 (0.69–1.13)	0.81 (0.62–1.05)	0.80 (0.62–1.03)	0.059	0.94 (0.86–1.02)
**Cardiovascular mortality (*n* = 140)**						
N of events/*n* of subjects	50/2473	28/2647	28/2583	34/2703	-	-
Person-years	27,204	29,341	28,721	29,851	-	-
Crude model	-1-	0.52 (0.33–0.82)	0.53 (0.33–0.84)	0.62 (0.40–0.96)	0.031	0.81 (0.69–0.95)
Model 1 (age, sex)	-1-	0.73 (0.46–1.17)	0.85 (0.53–1.36)	0.82 (0.53–1.27)	0.45	0.95 (0.82–1.10)
Model 1 + baseline disease/conditions	-1-	0.75 (0.47–1.20)	0.85 (0.53–1.36)	0.85 (0.55–1.32)	0.53	0.96 (0.83–1.12)
Model 1 + health behaviors	-1-	0.73 (0.46–1.17)	0.86 (0.53–1.37)	0.82 (0.53–1.28)	0.46	0.95 (0.81–1.09)
Model 1 + CSD, social factors and residence	-1-	0.78 (0.49–1.24)	0.91 (0.57–1.46)	0.88 (0.56–1.37)	0.67	0.97 (0.84–1.13)
Model 1 + all factors	-1-	0.77 (0.48–1.24)	0.90 (0.56–1.45)	0.89 (0.56–1.39)	0.68	0.97 (0.83–1.13)

Values are hazard ratios estimated with Cox regression and 95%CI. CSD = cumulative disadvantage score; social factors include marital status. Baseline diseases/conditions include: history of cancer, hyperlipidemia, hypertension, diabetes, and psychological assessment. Health behaviors include: smoking status, physical exercise, Mediterranean diet score, and body mass index.

**Table 3:** Hazard ratios (with 95% confidence interval) for all-cause and cardiovascular (CVD) mortality associated with domains of psychological resilience in the Moli-Sani Study cohort (*n* = 10,406), using data obtained from multiple imputation.

	All-Cause Mortality (*n* of Deaths = 478)		CVD Mortality (*n* of CVD Deaths = 140)	
*PR domains*	HR (95%CI)	*p*-value	HR (95%CI)	*p*-value
Factor 1 (positive acceptance of change)	0.89 (0.82–0.98)	**0.01**	0.88 (0.74–1.03)	0.11
Factor 2 (faith and hope)	0.99 (0.90–1.09)	0.87	1.12 (0.92–1.36)	0.25
Factor 3 (secure attachments and secure relationship)	1.05 (0.96–1.15)	0.32	1.14 (0.96–1.35)	0.13
Factor 4 (external locus of control and confidence in one’s ability)	1.06 (0.97–1.16)	0.23	0.98 (0.83–1.16)	0.83
Factor 5 (strengthening effects of stress and self-efficacy)	0.94 (0.86–1.03)	0.19	0.95 (0.80–1.12)	0.53

Values are hazard ratios estimated with Cox regression and 95%CI obtained from a multivariable-adjusted model including: age, sex, cumulative disadvantage score, marital status, residence, history of cancer, hyperlipidemia, hypertension, diabetes, psychological assessment, smoking status, physical exercise, Mediterranean diet score, and body mass index. Significant associations surviving Bonferroni correction for multiple testing (α = 0.01) are highlighted in bold.

**Table 4:** Sub-group analyses for the association of psychosocial resilience (1-SD increment in the CD-RISC) with all-cause and cardiovascular (CVD) mortality in the Moli-Sani Study cohort (*n* = 10,406), using data obtained from multiple imputation.

	All-Cause Mortality		CVD Mortality	
	N of Deaths/*n* of Subjects	HR (95%CI)	N of CVD Deaths/*n* of Subjects	HR (95%CI)
Study sample	478/10,406	0.94 (0.86–1.02)	140/10,406	0.97 (0.83–1.13)
Women	169/5236	0.89 (0.77–1.03)	55/5236	0.88 (0.69–1.12)
Men	309/5170	0.98 (0.88–1.09)	85/5170	1.06 (0.86–1.31)
*p* for interaction		0.17		0.066
Aged ≤ 65 year	213/9059	0.96 (0.84–1.10)	41/9059	1.40 (1.02–1.93)
Aged > 65 year	265/1347	0.92 (0.83–1.02)	99/1347	0.88 (0.74–1.04)
*p* for interaction		0.67		0.036
Low CSD	195/5956	0.97 (0.84–1.13)	50/5956	1.13 (0.84–1.53)
High CSD	283/4450	0.93 (0.84–1.03)	90/4450	0.92 (0.77–1.10)
*p* for interaction		0.41		0.21
Married	372/9051	0.95 (0.87–1.05)	98/9051	0.99 (0.83–1.20)
Unmarried	106/1355	0.89 (0.74–1.07)	42/1355	0.91 (0.69–1.21)
*p* for interaction		0.50		0.40
Low mental quality of life *	253/5203	0.97 (0.85–1.10)	77/5203	0.96 (0.76–1.21)
High mental quality of life *	225/5203	1.03 (0.91–1.18)	63/5203	1.13 (0.87–1.47)
*p* for interaction		0.40		0.42
Recruitment period 2005–2007	297/5788	0.97 (0.88–1.08)	90/5788	1.04 (0.86–1.25)
Recruitment period 2008–2010	181/4618	0.88 (0.75–1.02)	50/4618	0.89 (0.67–1.18)
*p* for interaction		0.47		0.66

Values are hazard ratios with 95%CI obtained from a multivariable model adjusted for: age, sex, education, housing tenure, occupational social class, residence, marital status, history of cancer, hyperlipidemia, hypertension, diabetes, psychological assessment, smoking status, physical exercise, Mediterranean diet score, and body mass index. * Mental quality of life measured by SF-36 and HRs were from the multivariable model further controlled for physical quality of life.

## Data Availability

The data underlying this article will be shared on reasonable request to the corresponding author. The data are stored in an institutional repository and access is restricted by the ethical approvals and the legislation of the European Union.

## Supplementary Materials

The following are available online at www.mdpi.com/article/10.3390/ijerph19010222/s1, Table S1: Baseline characteristics of the analytic sample vs excluded participants, Table S2: Factor loadings on the Moli-sani cohort (*n* = 10,396) CD-RISC items using a polychoric factor analysis, Table S3: Hazard ratios (with 95% Confidence Interval) for all-cause and cardiovascular mortality associated with domains of psychological resilience in different subgroups of the Moli-sani Study cohort (*n* = 10,406) using data obtained from multiple imputation. Supplementary Material File S1: list of Moli-sani study investigators.
