# Supplementary material for: Association of Psychological Resilience with All-Cause and Cardiovascular Mortality in a General Population in Italy: Prospective Findings from the Moli-Sani Study
Source: Int J Environ Res Public Health. 2021 Dec 25;19(1):222. doi: 10.3390/ijerph19010222 (PMC8750664; doi:10.3390/ijerph19010222)
Supplement: Supplementary file 1 [file ijerph-19-00222-s001.zip › ijerph-1491774-supplementary -modify.pdf]

Supplementary Table S1. Baseline characteristics of the analytic sample vs excluded participants

Characteristic	Analytic sample (n= 10,406)	Excluded participants (n = 6251)	p-value*
Women (%)	49.7	43.0	<0.0001
Age (mean, SD)	52.1 (10.5)	58.4 (11.9)	<0.0001
Baseline Cancer (%)	2.8	3.8	0.62
Baseline Diabetes (%)	3.2	5.1	0.47
Baseline Dyslipidaemia (%)	4.5	6.8	0.88
Baseline Hypertension (%)	20.7	30.8	0.48
Use of antidepressants (%)	3.0	3.2	0.95
Cumulative disadvantage score (mean, SD)	5.1 (1.8)	6.1 (1.7)	<0.0001
Current Smokers (%)	24.5	22.3	0.45
Leisure-time physical activity (mean, SD)	3.4 (3.8)	3.4 (4.2)	0.63
Mediterranean diet score (mean, SD)	4.3 (1.6)	4.4 (1.6)	0.97

*P value from logistic regression analysis controlled for age and sex.

Supplementary Table S2. Factor loadings on the Moli-sani cohort (n=10,396) CD-RISC items using a polychoric factor analysis

Factor1 (Positive Acceptance of Change)	Factor2 (Faith and Hope)	Factor3 (Secure Attachment and Secure Relationships)	Factor4 (External Locus of Control and Confidence in One's Abilities)	Factor5 (Strengthening Effects of Stress and Self-Efficacy)	Item	Item Description
**0.5108**	0.0756	0.1897	0.1332	0.2282	1	Able to adapt to change
0.1852	0.0576	**0.5875**	0.0943	0.0797	2	Close and secure relationships
0.0387	**0.5384**	0.2507	0.0458	−0.0685	3	Sometimes fate and God can help
**0.5845**	0.1114	0.1727	0.3149	0.2297	4	Can deal with whatever comes
**0.5326**	0.1659	0.2608	0.3336	0.1890	5	Past success gives confidence for new challenges
**0.5791**	0.1708	0.0842	0.1198	0.1629	6	See the humorous side of things
**0.5766**	0.1694	0.0408	0.2107	0.2295	7	Coping with stress strengthens
**0.4971**	0.2083	0.1058	0.1057	0.2106	8	Tend to bounce back after illness or hardship
0.1698	**0.4938**	0.0543	0.0192	0.0994	9	Things happen for a reason
0.3279	0.2320	0.1667	0.3395	0.2262	10	Best effort no matter what
0.3627	0.0273	0.1806	**0.4652**	0.4277	11	You can achieve your goals
0.2230	0.0594	0.2241	0.2211	**0.5559**	12	When things look hopeless. I don't give up
0.0852	0.2218	**0.6405**	0.1006	0.0908	13	Know where to turn for help
0.3566	0.0688	0.0854	0.3385	0.3524	14	Under pressure. focus and think clearly
0.2656	0.0966	−0.0888	0.2360	**0.4837**	15	Prefer to take the lead in problem solving
0.1793	0.1316	0.1668	0.0495	**0.6579**	16	Not easily discouraged by failure
0.3822	0.1292	0.0189	0.4549	**0.5047**	17	Think of self as strong person
0.3214	0.1140	0.0325	0.2325	**0.4608**	18	Make unpopular or difficult decisions
0.4146	0.2568	−0.0115	0.1614	0.3688	19	Can handle unpleasant feelings
0.1759	**0.4685**	−0.0587	0.1521	0.2089	20	Have to act on a hunch
0.1422	**0.5677**	0.1628	0.3173	0.1230	21	Strong sense of purpose
0.1952	0.2330	0.0111	**0.5384**	0.1225	22	In control of your life
0.3787	0.0418	0.0056	0.3929	0.3734	23	I like challenges
0.2526	0.0854	0.0947	**0.5017**	0.3523	24	You work to attain your goals
0.1222	0.1115	0.1651	**0.6072**	0.1194	25	Pride in your achievements

Each factor tags a different domain of psychological resilience. Factor 1 reflects positive acceptance of change (items 1, 4, 5, 6, 7, 8, Table 3); Factor 2 reflects faith and hope (items 3, 9, 20, 21); Factor 3 reflects secure attachment and secure relationships (items 2, 13); Factor 4 reflects external locus of control and confidence in

one's abilities (items 11, 22 ,24, 25); and Factor 5 reflects strengthening effects of stress and self-efficacy (items 12, 15, 16, 17, 18).

Supplementary Table S3. Hazard ratios (with 95% Confidence Interval) for all-cause and cardiovascular (CVD) mortality associated with domains of psychological resilience in different subgroups of the Moli-sani Study cohort (n=10,406) using data obtained from multiple imputation.

**Subjects Aged≤65 y (n = 9059)**	HR	Lower 95% CI	Upper 95% CI	p value
All-cause mortality (n=213)				
Factor 1	0.97	0.84	1.11	0.65
Factor 2	0.95	0.82	1.09	0.43
Factor 3	0.97	0.84	1.12	0.67
Factor 4	1.09	0.96	1.25	0.19
Factor 5	0.96	0.84	1.10	0.58
CVD mortality (n=41)				
Factor 1	1.24	0.89	1.72	0.20
Factor 2	1.42	1.03	1.95	0.03
Factor 3	1.19	0.85	1.66	0.32
Factor 4	0.82	0.59	1.12	0.21
Factor 5	1.22	0.89	1.67	0.21
**Subjects aged>65 y (n=1347)**				
All-cause mortality (n=265)				
Factor 1	0.85	0.75	0.95	0.01
Factor 2	1.06	0.92	1.22	0.42
Factor 3	1.09	0.97	1.23	0.16
Factor 4	1.04	0.92	1.17	0.57
Factor 5	0.90	0.79	1.02	0.11
CVD mortality (n=99)				
Factor 1	0.81	0.67	0.99	0.04
Factor 2	1.02	0.80	1.31	0.85
Factor 3	1.07	0.87	1.31	0.53
Factor 4	1.08	0.88	1.32	0.45
Factor 5	0.83	0.67	1.03	0.09
**Women (n=5236)**				
All-cause mortality (n=169)				
Factor 1	0.91	0.78	1.05	0.20
Factor 2	0.93	0.79	1.10	0.42
Factor 3	0.95	0.82	1.11	0.52
Factor 4	1.04	0.90	1.20	0.62
Factor 5	0.92	0.79	1.08	0.30
CVD mortality (n=55)				
Factor 1	0.97	0.75	1.26	0.84
Factor 2	0.99	0.73	1.36	0.97
Factor 3	0.92	0.69	1.22	0.56

Factor 4	0.95	0.74	1.22	0.68
Factor 5	0.79	0.59	1.04	0.09
**Men (n=5170)**				
All-cause mortality (n=309)				
Factor 1	0.90	0.80	1.01	0.07
Factor 2	1.04	0.92	1.17	0.57
Factor 3	1.10	0.98	1.23	0.11
Factor 4	1.07	0.96	1.20	0.24
Factor 5	0.95	0.85	1.07	0.39
CVD mortality (n=85)				
Factor 1	0.83	0.67	1.03	0.10
Factor 2	1.29	1.01	1.65	0.04
Factor 3	1.17	0.94	1.45	0.16
Factor 4	1.01	0.80	1.26	0.96
Factor 5	1.01	0.80	1.26	0.96
**Recruitment period 2005-2007 (n=5788)**				
All-cause mortality (n=297)				
Factor 1	0.87	0.78	0.98	0.02
Factor 2	1.08	0.96	1.22	0.22
Factor 3	1.10	0.97	1.23	0.13
Factor 4	1.06	0.95	1.19	0.28
Factor 5	0.93	0.83	1.04	0.22
CVD mortality (n=90)				
Factor 1	0.84	0.68	1.03	0.10
Factor 2	1.25	0.98	1.58	0.07
Factor 3	1.14	0.92	1.42	0.24
Factor 4	1.02	0.84	1.24	0.84
Factor 5	0.99	0.80	1.22	0.91
**Recruitment period 2008-2010 (n=4618)**				
All-cause mortality (n=181)				
Factor 1	0.92	0.80	1.07	0.28
Factor 2	0.84	0.71	0.99	0.04
Factor 3	0.99	0.85	1.14	0.85
Factor 4	1.05	0.89	1.22	0.58
Factor 5	0.96	0.81	1.12	0.57
CVD mortality (n=50)				
Factor 1	0.97	0.73	1.27	0.80
Factor 2	0.90	0.64	1.26	0.54
Factor 3	1.22	0.92	1.62	0.17
Factor 4	0.93	0.68	1.29	0.68
Factor 5	0.87	0.64	1.18	0.36

Values are Hazard ratios estimated with Cox regression and 95% CI obtained from a multivariable-adjusted model including age (continuous), sex, cumulative disadvantage score, marital status, residence, history of cancer, hyperlipidaemia, hypertension, diabetes, psychological assessment, smoking status, physical exercise, Mediterranean diet score and body mass index.
Factor 1 reflects positive acceptance of change (items 1, 4, 5, 6, 7, 8, Table 3); Factor 2 reflects faith and hope (items 3, 9, 20, 21); Factor 3 reflects secure attachment and secure relationships (items 2, 13); Factor 4 reflects external locus of control and confidence in one's abilities (items 11, 22 ,24, 25); and Factor 5 reflects strengthening effects of stress and self-efficacy (items 12, 15, 16, 17, 18)

Supplementary Materials: File S1

Moli-sani Study Investigators

The enrolment phase of the Moli-sani Study was conducted at the Research Laboratories of the Catholic University in Campobasso (Italy), the follow up of the Moli-sani cohort is being conducted at the Department of Epidemiology and Prevention of the IRCCS Neuromed, Pozzilli, Italy.

Steering Committee: Licia Iacoviello*°(Chairperson), Giovanni de Gaetano* and Maria Benedetta Donati*.

Scientific secretariat: Marialaura Bonaccio*, Americo Bonanni*, Chiara Cerletti*, Simona Costanzo*, Amalia De Curtis*, Augusto Di Castelnuovo§, Francesco Gianfagna°§, Mariarosaria Persichillo*, Teresa Di Prospero* (Secretary).

Safety and Ethical Committee: Jozeph Vermylen (Catholic University, Leuven, Belgio) (Chairperson), Ignacio De Paula Carrasco (Accademia Pontificia Pro Vita, Roma, Italy), Antonio Spagnuolo (Catholic University, Roma, Italy).

External Event adjudicating Committee: Deodato Assanelli (Brescia, Italy), Vincenzo Centritto (Campobasso, Italy).

Baseline and Follow-up data management: Simona Costanzo* (Coordinator), Marco Olivieri (Associazione Cuore Sano, Campobasso, Italy), Teresa Panzera*.

Data Analysis: Augusto Di Castelnuovo§ (Coordinator), Marialaura Bonaccio*, Simona Costanzo*, Simona Esposito*, Alessandro Gialluisi*, Francesco Gianfagna°§, Emilia Ruggiero*.

Biobank and biochemical laboratory: Amalia De Curtis* (Coordinator), Sara Magnacca§.

Genetic laboratory: Benedetta Izzi* (Coordinator), Annalisa Marotta*, Fabrizia Noro*, Roberta Parisi*, Alfonsina Tirozzi*.

Recruitment staff: Mariarosaria Persichillo* (Coordinator), Francesca Bracone*, Francesca De Lucia (Associazione Cuore Sano, Campobasso, Italy), Cristiana Mignogna°, Teresa Panzera*, Livia Rago*.

Communication and Press Office: Americo Bonanni*.

Regional Health Institutions: Direzione Generale per la Salute - Regione Molise; Azienda Sanitaria Regionale del Molise (ASReM, Italy); Molise Dati Spa (Campobasso, Italy); Offices of vital statistics of the Molise region.

Hospitals: Presidi Ospedalieri ASReM: Ospedale A. Cardarelli – Campobasso, Ospedale F. Veneziale – Isernia, Ospedale San Timoteo - Termoli (CB), Ospedale Ss. Rosario - Venafro (IS), Ospedale Vietri – Larino (CB), Ospedale San Francesco Caracciolo - Agnone (IS); Casa di Cura Villa Maria - Campobasso; Ospedale Gemelli Molise - Campobasso; IRCCS Neuromed - Pozzilli (IS).

*Department of Epidemiology and Prevention, IRCCS Neuromed, Pozzilli, Italy

°Department of Medicine and Surgery, University of Insubria, Varese, Italy
§Mediterranea Cardiocentro, Napoli, Italy
Baseline Recruitment staff is available at https://www.moli-sani.org/?page_id=173
